# Personalized Medicine in Medullary Thyroid Carcinoma: A Broad Review of Emerging Treatments

**DOI:** 10.3390/jpm13071132

**Published:** 2023-07-13

**Authors:** Rui Sousa Martins, Tito Teles Jesus, Luís Cardoso, Paula Soares, João Vinagre

**Affiliations:** 1 Instituto de Investigação e Inovação em Saúde (i3S), Universidade do Porto, 4200-135 Porto, Portugal; rmartins@i3s.up.pt (R.S.M.); tjesus@ipatimup.pt (T.T.J.); lcardoso@i3s.up.pt (L.C.); psoares@ipatimup.pt (P.S.); 2 Instituto de Patologia e Imunologia Molecular da Universidade do Porto (Ipatimup), 4200-135 Porto, Portugal; 3 Faculdade de Ciências da Universidade do Porto (FCUP), 4169-007 Porto, Portugal; 4 Departamento de Endocrinologia, Diabetes e Metabolismo do Centro Hospitalar Universitário de Coimbra, 3000-075 Coimbra, Portugal; 5 Faculdade de Medicina da Universidade do Porto (FMUP), 4200-319 Porto, Portugal

**Keywords:** MTC, *RET*, signaling pathways, TKIs, multikinase inhibitors, *RET*-specific inhibitors

## Abstract

Medullary thyroid carcinoma (MTC) arises from parafollicular cells in the thyroid gland, and although rare, it represents an aggressive type of thyroid cancer. MTC is recognized for its low mutational burden, with point mutations in *RET* or *RAS* genes being the most common oncogenic events. MTC can be resistant to cytotoxic chemotherapy, and multitarget kinase inhibitors (MKIs) have been considered a treatment option. They act by inhibiting the activities of specific tyrosine kinase receptors involved in tumor growth and angiogenesis. Several tyrosine kinase inhibitors are approved in the treatment of advanced MTC, including vandetanib and cabozantinib. However, due to the significant number of adverse events, debatable efficiency and resistance, there is a need for novel RET-specific TKIs. Newer RET-specific TKIs are expected to overcome previous limitations and improve patient outcomes. Herein, we aim to review MTC signaling pathways, the most recent options for treatment and the applications for personalized medicine.

## 1. Introduction

Medullary thyroid carcinoma (MTC) is a rare and aggressive thyroid cancer that arises from the parafollicular cells of the thyroid gland, also referred to as C cells. C cells are neuroendocrine cells that specialize in secreting the hormone calcitonin, which is involved in the regulation of calcium metabolism [1]. MTC accounts for 2–4% of all thyroid cancers, with most being sporadic. Approximately 25% of cases are caused by an inherited genetic disorder, i.e., multiple endocrine neoplasia type 2 (MEN2) syndrome: familial MTC (FMTC), MEN2A, or MEN2B [2,3]. Patients with an FMTC subtype of MEN2 present only with isolated MTC, while patients with MEN2A typically present with MTC at a variable age (i.e., depending on the mutation) associated with pheochromocytoma and/or parathyroid adenoma. Other features of MEN2A include cutaneous lichen amyloidosis and Hirschprung’s disease, and again, the phenotypic expression of the latter features depends upon the causative mutation. MEN2B is characterized by the presence of MTC and an increased risk for pheochromocytoma, a physical appearance of marfanoid habitus, the presence of multiple neuromas of the oral mucosa, gastrointestinal ganglioneuromatosis and medullated corneal nerves [4]. Overall, the main differences between these subtypes lie in the specific combinations of tumors and accompanying features, with MEN2B presenting the most severe clinical course. MEN2 syndrome, due to mutations in the RET proto-oncogene (*RET*), presents a well-described genotype–phenotype association, so genetic testing is pivotal for MEN2 subtype diagnosis, MTC risk estimation, adequate management and the monitoring and follow-up of patients in order to improve outcomes for affected individuals [5,6,7,8]. Furthermore, ~5–10% of cases of apparently sporadic MTC harbor a germline mutation on *RET* [5,6,7,8]. The prevalence of hereditary syndromes in MTC (~25%) is higher when compared to other cancers in which the hereditary forms represent 10% or lower, e.g., in melanoma, hereditary cases account for 10% [9].

The genotyping of these patients is crucial as surgery is the only curative treatment, and prophylactic thyroidectomy in childhood is advised for children who harbor some germline pathogenic variants of *RET* [10]. Treatment options for recurrent or metastatic MTC may include surgery, radiotherapy and systemic therapies [4]. The 10-year disease-specific mortality varies from ~15 to 40%, depending on the stage and age at diagnosis. However, when detected in an early stage (i.e., intrathyroidal tumors), MTC has a much higher survival rate (i.e., ~90% at 35 years), reflecting the paramount importance of early diagnosis [11,12]. MTC frequently spreads to lymph nodes even in smaller tumors, and at diagnosis, ~50–75% of patients already present with lymph node metastases [13,14,15]. Therefore, more effective and targeted therapies are needed to improve patients’ clinical outcomes. Tyrosine kinase inhibitors (TKIs) are some of these targeted therapeutic modalities; they have been under the spotlight and are approved by the United States Food and Drug Administration (FDA) for the treatment of advanced MTC. TKIs were shown to suppress the function of the RET receptor, including in children with MEN2 hereditary syndrome [16,17]. Recent trials evaluated the possibility of treating MTC by combining TKIs with other targeted therapies, mainly due to the adverse events associated with TKIs and the need for more personalized medicine [18]. TKIs, e.g., vandetanib and cabozantinib, have offered novel possibilities for treating patients with advanced MTC but are still hampered due to their mild efficiency and substantial adverse events due to the setting of MKIs. We are now entering a new era of novel RET-specific TKIs that are already FDA approved for the treatment of other diseases and are expected to overcome previous limitations. In this review, we aim to review the emerging treatments for MTC, with particular focus on RET-specific TKIs, which are paving the way to personalized medicine.

## 2. Signaling Pathways in MTC

### 2.1. RET and Its Downstream Signaling Cascades

RET is expressed in cells originating from the neural crest, branchial arches, and urogenital system [19,20]. *RET* is located in the long arm of chromosome 10 (10q11.2) and was identified for the first time by Takahashi et al.; it was named “REarranged during Transfection” as it is able to undergo activation following genetic rearrangement [21]. *RET* encodes two main alternative transcript isoforms, RET9 and RET51, which differ in their C-terminal, to produce a 150 kD receptor, tyrosine kinase [22]. The RET protein is characterized by three distinct functional domains: (1) an extracellular domain containing four cadherin-like repeats that mediate receptor oligomerization and a cysteine-rich region involved in intraprotein disulfide bond formation; (2) a transmembrane domain; (3) and an intracellular domain that incorporates several tyrosine residues necessary for the activity of RET protein kinase. The different forms of tyrosine kinase receptors resulting from the *RET* gene are essential for cell proliferation and differentiation [4,23].

**Figure 1:**
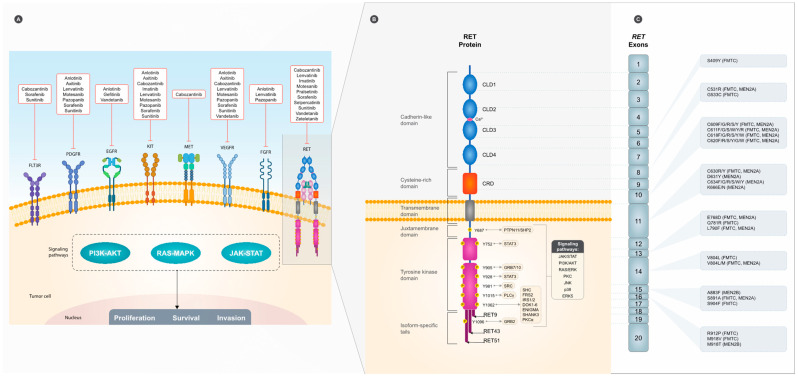
Schematic representation of tyrosine kinase receptors (**A**): the vascular endothelial growth factor receptor (VEGFR), epidermal growth factor receptor (EGFR), platelet-derived growth factor receptor (PDGFR), fibroblast growth factor receptor (FGFR) and RET proto-oncogene receptor (RET) are the most important receptors involved in medullary thyroid carcinoma tumorigenesis. The inhibitors of each receptor are listed in the accompanying boxes. Vandetanib and cabozantinib are two U.S. Food and Drug Administration (FDA)- and European Medicines Agency (EMA)-approved inhibitors used in MTC treatment. Selpercatinib and pralsetinib represent the new generation of inhibitors and are specific to the RET protein. (**B**): The RET protein is composed of an extracellular domain containing four cadherin-like domains (CLD1, CLD2, CLD3 and CLD4) responsible for receptor oligomerization and a cysteine-rich domains (CRD) involved in intraprotein disulfide bond formation. The intracellular domain contains several tyrosine residues necessary for the activation of different signaling pathways; different pathways activated by the RET protein are represented, and the link to the tyrosine residues is also shown. Y1096 is unique and present only in the *RET51* long isoform. Y687 recruits and binds SHP2 phosphatase, inducing the activation of PI3K/AKT [99]. Y752 and Y928 in constitutively active RET are relevant docking sites for signal transducer and activator of transcription 3 (STAT3) [25,33]. Y905 has been identified as docking site for Grb7 [100], Grb10 [101] and SH2B1β [102]. Y981 is critical for binding and activation of Src kinase [34]. Y1015 is the docking site for PLC-γ [35], and it also mediates migratory signals [103]. Y1062 regulates the binding of several adaptor proteins such as SHC adaptor protein (SHC) family members, IRS1/2, Dok1, Dok4/5, Dok6, Enigma, PKCα and Shank3 [36,37,38,39,40,41,42,43,44,45,46,47,48,49,50]. (**C**) A schematic representation of *RET* 20 exons: the mutations and corresponding phenotypes are also shown. On the right are listed the mutations, the exon at which they occur and the corresponding disease (if available in the ARUP database—http://arup.utah.edu/database/MEN2/MEN2_welcome.php (accessed on 23 May 2023).

RET is activated upon the fulfilment of multiple events, including the binding of Ca^2+^ ions to the cadherin-like domains [24] and the binding of glial cell line-derived neurotrophic factor (GDNF) family ligands (GFL) to glycosyl phosphatidylinositol-anchored co-receptor GDNF-family receptor-α (GFRα1-4) proteins, which recruit two RET receptors into close proximity and allow for the dimerization and autophosphorylation of several residues on the cytoplasmic tails, which may occur in 14 of the 18 residues [25,26,27]. The phosphorylation of these tyrosine residues within the RET intracellular enzymatic domain results in changes in RET protein conformation, enabling the binding of one or more cytoplasmic adaptor proteins that subsequently activate downstream cytoplasmic signaling pathways [28,29,30]. RET activates numerous intracellular signaling cascades that are implicated in the regulation of cell survival/apoptosis, differentiation, proliferation, migration, chemotaxis, branching morphogenesis, neurite outgrowth and synaptic plasticity [29,30]. RET cytoplasmic tyrosine-phosphorylated residues recruit several adaptor proteins essential to the signal propagation of external stimuli and to trigger downstream signaling cascades, namely, RAS/RAF/MEK/ERK, PI3K/AKT and JAK2/STAT3, and phospholipase C-γ (PLC-γ) pathways [28,29,30]. The most-studied specific functional implications were from the phosphorylation at the tyrosine residues Y905, Y981, Y1015, Y1062 and Y1096 [31,32]. Y905 stabilizes the conformation of RET kinase and promotes the autophosphorylation of tyrosine residues mainly located on the C-terminal tail [25,33]. Y981 is critical for the binding and activation of Src kinase to mediate pro-survival signaling [34]. Y1015 is the docking site for PLC-γ, which activates the protein kinase C (PKC) pathway [35]. Y1062 is present at the C-terminal of RET and regulates the binding of several adaptor proteins, such as SHC adaptor protein (SHC) family members, IRS1/2, Dok1, Dok4/5, Dok6, Enigma, PKCα and Shank3 [36,37,38,39,40,41,42,43,44,45,46,47,48,49,50]. Y1096 is unique and is only present in the RET51 isoform. The activation of these downstream signaling cascades (MAPK/ERK and PI3K/AKT) is essential for cell survival, differentiation, proliferation and motility [51,52,53]. The role of additional tyrosine residues (Y826 and Y1029) that are phosphorylated upon binding to GFLs in RET signaling remain to be disclosed. A summary of RET protein domains and interactions is depicted in Figure 1.

### 2.2. MAPK Activation (RAS/RAF/MEK/ERK)

The binding of RET receptor tyrosine kinase (RTK) to GDNF (or persephin, in the case of C-cells) is recognized mainly via the activation of two significant pathways: the MAPK and the PI3K/AKT/mTOR pathways [54]. MAPK leads to a wide number of downstream signaling cascades, of which RAS/RAF/MEK/ERK represents one of the most commonly altered cascades in human cancers [55]. Once RAS is activated, it stimulates several intracellular transducers such as RAF and PI3K, among others, to regulate proliferation, survival and differentiation [56]. Three human *RAS* genes, including *H-RAS*, *N-RAS*, and *K-RAS,* are responsible for controlling the intracellular signaling cascade involved in a number of crucial cellular functions, such as cell division, adhesion, migration, and apoptosis [55]. In the thyroid, *RAS* oncogenic modifications have been proposed as some of the first tumorigenic events [57]. In two-thirds of sporadic cases of MTC, somatic *RET* mutations are detectable. Recently, some studies have demonstrated a high prevalence of *RAS* somatic mutations in *RET*-negative tumors [58,59,60]. Indeed, in patients with sporadic MTC, between 12.6% and 40.9% harbor *RAS* mutations [58,61,62], and 68% of *RET*-negative MTC patients carry *RAS* mutations in comparison to 2.5% of *RET*-positive MTC patients [58]. Our experience tends toward the mutually exclusivity of *RAS* and *RET* mutation, although it is a frequent event in somatic cases [61]. Similar results were obtained via the application of novel technologies, i.e., exome sequencing, by Agrawal et al. [59].

The activation of *RAS* or *RAF* can cause the production and release of a protein that mediates differentiation and G1 cell cycle arrest in MTC cells. This protein was identified as the leukemia inhibitory factor (LIF) via protein purification and mass spectrometry [63]. LIF-mediated growth arrest and differentiation in MTC cells requires STAT3 activation. Additionally, the RAS/RAF/MEK/ERK pathway may mediate growth arrest and differentiation via a second mechanism unrelated to LIF/JAK/STAT3. This unique autocrine–paracrine mechanism defines a novel mode of inhibiting RAS/RAF-induced cell growth by mediating crosstalk between the RAS/RAF/MEK/ERK and JAK-STAT pathways [63,64]. RAF-1’s specific role in MTC was studied by Kunnimalaiyaan et al., who established the pathway’s importance by inhibiting TT cells both in vivo and in vitro, using lithium chloride in TT xenograft mice. This led to the inhibition of GSK3B, which is a product of RAF-1 phosphorylation, resulting in a significant decrease in tumor volume and revealing the important role of this downstream signaling cascade in MTC [65].

### 2.3. PI3K/AKT/mTOR Activation

The PI3K/AKT/mTOR signaling pathway is a central signaling cascade that is involved in critical cellular functions, and it is important in tumorigenesis because it promotes cell growth and proliferation [66]. Upon its recruitment to the plasma membrane by activated RTK, PI3K phosphorylates PIP3, which in turn activates downstream RAC small GTPase, PDK1 and AKT (also known as PKB) [67]. AKT phosphorylates and inactivates pro-apoptotic transcription factors [67]. AKT also activates serine/threonine kinase mTOR. mTOR is associated with two complexes: the mTORC1 complex, which is rapamycin-sensitive and leads to S6K1 and eIF4E, which are essential transcription factors that increase cell growth and proliferation [68]; and the mTORC2 complex [69]. mTORC1 also contributes to NF-κB activation (NF-κB promotes the transcription of genes involved in cell proliferation, angiogenesis, metastasis and inflammation, as well as the repression of apoptosis) [70]. *RET* leads to NF-κB activation via the phosphorylation and degradation of NF-κB’s inhibitors. This pathway is constitutively active in MTC [71].

Numerous studies have provided evidence of the dysregulation of the PI3K/AKT/mTOR signaling cascade in MTC [61,72,73,74]. Rapa et al. did not identify *PI3K* mutations but showed that the mTOR intracellular signaling pathway is functionally activated in patients with MTC and positively correlated with the presence of germline *RET* mutations [73]. In Tamburrino et al.’s study, the PI3K/AKT/mTOR pathway was reported to be activated in MTC and metastatic lymph nodes. This pathway was shown to sustain malignant features of different MTC cell models [72]. The activation of mTOR signaling was demonstrated in 96–100% of patients affected by MTC, according to the studies by Tamburrino et al. [72] and Kouvaraki et al. [75]. In our experience, we observed that *RAS* mutations were significantly associated with a higher intensity of p-S6 expression, particularly in invasive tumors or in MTCs with lymph node metastases, which is suggestive of mTOR pathway activation in this setting [61].

## 3. RET Mutations in MTC

*RET* mutations can be found in more than 95% of cases of hereditary MTC and in up to 65% of cases of sporadic MTC [5,76]. In MEN2A, the most frequently occurring mutations are present in exon 10 (codons 609, 611, 618 and 620) or exon 11 (codons 630 and 634). Particularly, the mutations involving exon 11 (codon 634) represent ~87% of all *RET* mutations implicated in MEN2A, with C634R (52.5%) and C634Y (25.4%) being the most frequent events [77,78]. MEN2B is commonly associated with mutations in the tyrosine kinase domains in exons 15 and 16 (codons 883, 891 and 918), and M918T is recognized as the highest risk mutation for MTC, followed by C634 and A883F. Tumors with mutations involving C634 and M918T are also associated with more aggressive behavior in multiple endocrine neoplasia type 2 A (MEN2A) and MEN2B; mutations involving C634 and V804M are also associated with cutaneous lichen amyloidosis, while mutations involving codons 609, 611, 618 and 620 have been associated with Hirschsprung’s disease. In FMTC, mutations in exons 8, 10, 11, 13, 14 and 15 partially overlap with the mutations found in MEN2A [79,80,81,82,83,84,85]. In Figure 1, the most common *RET* germline mutations described in the literature and the associated phenotypes, if reported, are presented. Most cases of MTC are sporadic and non-familial, and approximately half of the cases have *RET* somatic mutations, with M918T being the most frequent one [86,87].

The mutation type appears to also have genotype–phenotype implications. A *RET* mutation in a MEN2A (C634W) patient was reported to activate PI3K and its downstream effector AKT [88]. It was also demonstrated that the mutation of Y1062 (the docking site for Shc and Enigma on RET) abolishes the transforming activity of *RET*-MEN2A by abrogating the binding of PI3K to RET and the following activation of the PI3K/AKT pathway [37]. *RET*-MEN2B (RET M918T) is more efficiently autophosphorylated at RET Y1062 than *RET*-MEN2A, leading to increased constitutive activation of the MAPK and PI3K/AKT/mTOR signaling pathways [89]. Apart from functionality, genotyping may play a crucial role in the choice of advanced therapeutic modalities. Genotyping is a predictor of response to vandetanib and cabozantinib (which will be addressed in further detail) since patients with an M918T mutation presented with a greater response to vandetanib in comparison with M918T-negative patients (54.5% vs. 32%) [90]. In another trial, cabozantinib offered longer PFS in M918T-positive patients as well. The objective response rates (ORRs) for *RET*-mutation-positive cases, *RET*-mutation-negative cases and *RAS*-mutation-positive cases were 32%, 22% and 31%, respectively [91]. In vitro, Carlomagno et al. verified that *RET* codon 804 and 806 mutations confer resistance to vandetanib treatment [92].

## 4. Treatments Available to Current MTC Patients

### 4.1. Multitarget Kinase Inhibitors (MKIs)

In recent years, a significant amount of effort has been put into finding novel and efficient treatment options for tumors dependent on tyrosine kinase cascades. The most common receptors involved in malignancies include RET receptors, vascular endothelial growth factors (VEGFR-2 and VEGFR-3) and the epidermal growth factor receptor (EGFR), whose overexpression is observed in metastatic MTC [93]. VEGF, platelet-derived growth factor (PDGF), fibroblast growth factor (FGF), and angiopoietins, which act by binding to tyrosine kinase receptors on endothelial and other stromal cells, initiate angiogenesis [94]. Many tumor and host cells express VEGF, which is also upregulated in the tumor microenvironment [95]. The VEGF receptors VEGFR-1, VEGFR-2 and VEGFR-3 bind to various members of the VEGF family (VEGF-A, VEGF-B, VEGF-C and VEGF-D), and the main tyrosine kinase receptor, VEGFR-2, is responsible for regulating several processes such as vascular permeability and endothelial cell proliferation, invasion, migration and survival [96]. Additional receptors, such as PDGF receptors (PDGFR), that control pericyte differentiation and vascular survival are expressed on a variety of cell types, including pericytes and smooth muscle cells [97], and in some tumor cells, e.g., glioblastomas, PDGFR functions as an autocrine growth and survival signal [98]. These characteristics enhance the importance of disrupting these malignant pathways in cancer treatment. In Figure 1, oncogenic signaling pathways in MTC and their respective drug targets are illustrated.

In MTC, several MKIs are available and have been subjected to clinical trials, see Table 1.

Vandetanib is the most frequently prescribed. It obtained FDA approval in April 2011 for the treatment of patients with symptomatic or progressive MTC who presented with unresectable, locally advanced or metastatic disease. It showed an increase in the 6-month progression-free survival (PFS) of 83% of vandetanib-treated patients in comparison to 63% of placebo-treated patients, but study inclusion was not restricted to patients with *RET* mutations [90]. In the 298 sporadic MTC patients included in this trial, 155 patients had *RET* mutations, 8 patients did not have *RET* mutations and the *RET* mutation status of 135 patients was not disclosed [90].

Cabozantinib is also an FDA-approved MKI that is responsible for the inhibition of hepatocyte growth receptor (MET), VEGFR2 and RET. In comparison to the placebo, the estimated median PFS for cabozantinib was 11.2 months, as opposed to 4.0 months in the placebo group (95% CI, 0.19–0.40; *p* = 0.001). PFS increased in all subgroups of patients, including age, prior TKI treatment and *RET* mutational status (hereditary or sporadic), treated with cabozantinib. Independent of *RET* mutation status, cabozantinib had a response rate of 28%, whereas the placebo had no response rate (0%) [104]. Another trial with a minimum follow-up of 42 months that compared cabozantinib to a placebo revealed a non-statistically significant increase of 5.5 months in the median overall survival (OS)—26.6 vs. 21.1 months, respectively [112]. However, for patients carrying the M918T mutation, the OS was significantly increased to 44.3 months when compared to 18.9 months for those treated with a placebo. Regarding the pediatric cases, both drugs (i.e., vandetanib and cabozantinib) have been used with similar outcomes as in adults [113,114]. Despite the presence of more side effects, none of the patients needed to discontinue the treatment; the side effects included bleeding, weight loss, hypertension, hepatotoxicity, renal toxicity, pulmonary toxicity and gastrointestinal disturbances (diarrhea or constipation).

Sorafenib was the first MKI produced in the field of oncology. Until its approval, two other agents, erlotinib and imatinib, which target EGFR and BCR-ABL, had already shown clinical benefits [115,116,117]. Sorafenib inhibits RAF-1, CRAF and BRAF kinases, pro-angiogenic receptor tyrosine kinases (RTKs), VEGFR 1/2/3, PDGFRβ, FGFR1 and other RTKs involved in tumorigenesis (KIT, FLT3 and RET) [118]. In vitro, Carlomagno et al. used sorafenib to treat TPC1 and TT thyroid cell lines harboring *RET* mutations and RAT1 fibroblasts transformed via oncogenic *RET* mutants, leading to tumor shrinking and a decrease in RET phosphorylation [119]. In clinical trials, Lam et al. reported the treatment of 16 sporadic MTC (arm B) patients with sorafenib. One of the 16 patients in arm B who received treatment presented with partial response (PR); the other 14 had stable disease (SD: 87.5%; 95% CI, 61.7–99.5%), and the last patient was non-evaluable for RECIST response due to progressing disease (PD). One patient had a PR of +21 months, four patients had SD for 15 months, four patients had SD for 6 months, and one patient had PD in a post-hoc examination of the ten patients in arm B who had PD prior to research; within this group, 17.9 months was the median PFS. Most patients experienced a decrease in tumor markers (i.e., calcitonin and carcinoembryonic antigen), and 10 of the 12 spontaneous MTCs examined had *RET* mutations. The most frequent adverse events (AEs) were diarrhea, rash and hypertension. Despite the rarity of significant AEs, one death was reported [105].

Sunitinib is an oral small molecular MKI that exhibits potent anti-angiogenic and anti-tumoral activities. This MKI was purposefully created and selected due to its high bioavailability and nanomolar-range effectiveness against the anti-angiogenic RTKs, PDGFR and VEGFR. It can be used to treat small-cell lung cancer, GISTs, breast cancer, acute myelogenous leukemia and MTC due to the inhibition of tyrosine kinases such as KIT, FLT3, colony-stimulating factor 1 (CSF-1) and RET. Its clinical efficiency has been demonstrated in neuroendocrine, colon, and breast cancers in phase II trials, whereas its definitive efficiency was demonstrated in advanced renal cell carcinoma and in imatinib-refractory GISTs, leading to the FDA approval of sunitinib for the treatment of these two diseases [120]. The success demonstrated in both these tumor models led to the development of trials and more recent studies on the utility of sunitinib for locally advanced metastatic MTC. A study by Kuhar et al. used sunitinib treatment in 20 patients out of 30 patients with MTC: the median PFS on TKI was 10.6 months (95% CI 7.1–14), whereas the median PFS on chemotherapy was 3.5 (95% CI 1.4–5.5) months. The median OS from diagnosis was 38.2 months (95% CI 4.7–71.7), and the median OS from metastasis presentation was 20.9 months (95% CI 13.8–27.9). Due to incurable locally advanced and metastatic MTC, eight patients (five females and three males; 58–86 years of age, median age 70 years) received induction TKI treatment. Two patients presented with SD, whereas the other two presented with PD (25%); four patients experienced AEs of grade 3 or 4 (two neutropenia, one thrombocytopenia and one diarrhea). The response rate to induction TKI was 50%. Regarding AEs, fatigue, hypertension, dysgeusia and cutaneous papules were the most frequent toxicities reported in more than half of the patients [106]. Carr et al. investigated the efficacy and safety of daily sunitinib treatment in patients with advanced MTC as the disease is usually challenging to manage [121]. The study found that sunitinib treatment resulted in a disease control rate (DCR) of 77%, and 17% of patients presented with progression; only one patient had complete response (CR) [121]. AEs due to sunitinib included fatigue, neutropenia, hand/foot syndrome, diarrhea and leukopenia. One patient on anti-coagulation therapy died of gastrointestinal bleeding.

Lenvatinib is an MKI of RET, PDGFR, VEGFR, FGFR and KIT. Its effectiveness was demonstrated in locally recurring or metastatic, progressive, radioactive iodine-refractory differentiated thyroid cancer [107]. In a study involving lenvatinib, an analysis of PFS revealed that in comparison to 3.6 months (95% CI, 2.2–3.7) for the placebo group, the median PFS with lenvatinib was increased to 18.3 months; 202 patients had PD (93 patients in the lenvatinib group and 109 patients in the placebo group), and 18 patients died (14 in the lenvatinib group and 4 in the placebo group). Despite the PFS success rate, the AEs and death rate among the lenvatinib-treated group could not be disregarded: the treatment-related AEs of any grade, which occurred in more than 40% of patients in the lenvatinib group, were hypertension (67.8%), diarrhea (59.4%), fatigue or asthenia (59.0%), decreased appetite (50.2%), decreased weight (46.4%) and nausea (41.0%). Exclusions occurred during the study because of drug-related AEs in 37 patients who received lenvatinib (14.2%) and 3 patients who received the placebo (2.3%). In the lenvatinib group, 6 out of 20 deaths that occurred during the treatment period were considered to be drug-related [107]. Masaki et al. evaluated lenvatinib in 16 patients with thyroid cancer, including MTC, for tumor size reduction: 13 patients showed a ≥10% decrease in tumor size within 8 weeks, 2 with ≥10% over 8 weeks and 1 with no reduction; a reduction in tumor size leads to other treatment possibilities, mainly surgery [122]. More recently, Matrone et al. evaluated lenvatinib in advanced metastatic MTC. Because each patient had different cancer spread patterns, this team performed an analysis of different metastatic sites, such as the lymph nodes, bones, lungs and liver. This study was performed in ten patients: nine received treatment for more than 6 months, and the remaining one discontinued lenvatinib due to respiratory failure after 3 months and died before the first evaluation. At the last evaluation (median 12 months (IQR 9–31), range 7–31 months), seven patients presented with SD. PR and PD were observed in one patient each. Five patients died due to respiratory distress, neoplastic cachexia or hepatic failure, and three patients started therapy with different TKIs: cabozantinib and selpercatinib. [123].

With wider usage, anlotinib is an MKI used in many types of cancers, such as non-small-cell lung cancer (NSCLC), osteosarcomas and endometriomas [124,125]. The receptors that anlotinib targets are PDGFR, KIT, FGFR, VEGFR and EGFR [126]. A clinical trial was performed using anlotinib in 91 MTC patients. Of the patients, 89% a history of surgical treatment, 26.4% had undergone radiotherapy previously and 16.5% had undergone chemotherapy [108]. PFS was the main outcome observed in this trial. The ORR, i.e., the total number of patients with CR and PR, was one of the secondary objectives. The ORR of anlotinib treatment was 48.4%. The median PFS was significantly higher in the anlotinib group than in the placebo group (20.7 months vs. 11.1 months, *p* = 0.029). Given the long treatment course, safety is crucial when using TKI therapy for MTC. Anlotinib was tolerated excellently and demonstrated lower toxicity in this trial. Most AEs were grades 1–2 and were treatable. Diarrhea, hypertension, increased lipase, hypertriglyceridemia and palmar–plantar erythrodysesthesia syndrome were high-grade (3–4) treatment-related AEs that occurred in >5% of patients receiving anlotinib. This study did not evaluate *RET* mutational status, so the impact of *RET* gene status on anlotinib treatment response remains uncharacterized.

Axitinib is an anti-angiogenic MKI. This type of TKI is used to treat radioactive iodine-refractory differentiated thyroid cancer or MTC refractory to standard surgery or locoregional therapies or locoregional therapies. Unlike other MKIs, e.g., lenvatinib, axitinib offered substantially better results when used as first-line treatment. A clinical trial by Capdevila et al. found a difference between the ORRs of first- and second-line treatments, and better outcomes were reported with the use of axitinib as a first-line treatment, with an ORR of 53% and a median PFS of 13.6 months compared with 16.7% and 10.6 months as a second-line treatment [109]. Cohen et al. evaluated 11 stage IV MTC patients submitted to axitinib, and SD was observed in three patients and PR in two. Patients who demonstrated a response also showed a decrease in serum calcitonin concentration, specially the two patients with PR. The most common AEs were fatigue, diarrhea, nausea, anorexia, hypertension, stomatitis, weight loss and headache [127].

Pazopanib is a small-molecule inhibitor of kinases, including VEGFR-1, -2, and -3, PDGFR-α and -β, FGFR and KIT. It also inhibits other kinases, including RET, but it inhibits RET 280 times less than VEGFR [128]. Bible et al. conducted a clinical trial evaluating pazopanib for targeted medicine. In previous studies, they reported ORRs of 49% in patients with DTC and 0% in patients with advanced anaplastic thyroid cancer. In this trial, they tested pazopanib’s effects in two cell lines, BHP2-7 (DTC) and TT MTC cells. Compared to controls, pazopanib treatment significantly lowered proliferation in each cell line (*p* < 0.001), but its impact on TT vs. BHP2-7 cells was not statistically different. This finding indicated the potential usage of pazopanib in both DTC and MTC, leading to a clinical trial which included 35 patients (28 men and 7 women) aged 26 to 83 years (median age: 60 years). The lymph nodes (80%), liver (57.1%), bone (51.4%) and lung (45.7%) were the most frequent locations of metastases. Radiation therapy (42.8%) and systemic therapy (42.9%) were previously used. There were five PRs (14.3%, 90% CI 5.8–27.7%) among the 35 registered patients which lasted for 29 weeks, 1 year, 1.8 years and 4.0+ years, respectively. At the time of the data lock, eight patients (23%) were still living without PD, five were alive with PD and 22 had died as a result of illness. One of these 22 fatalities was thought to be linked to pazopanib. The median PFS and OS times were estimated to be 9.4 months and 19.9 months, respectively. More than 70% of the participants remained on the study therapy for more than 6 months. Despite the improvements regarding PFS and OS, there were clear AEs such as hypertension, fatigue, diarrhea and abnormal liver tests. Three out of thirty-five patients (8.6%) ceased taking the medication due to AEs. One patient died after withdrawing from the experiment, which was considered potentially treatment-related [110].

Motesanib is an MKI of VEGFR-1, -2 and -3 and PDGFR, KIT and RET. In *RET*-mutated cases, this drug did not demonstrate clinical improvement. Schlumberger et al. conducted a clinical trial with 91 MTC patients receiving motesanib [111]. Two (2%) of these patients obtained an objective response (95% CI, 0.3% to 7.7%); their response durations were 32 weeks and 21 weeks, respectively. Approximately 80% of patients attained SD (48% had durable SD ≥ 24 weeks), 8% had PD and 9% were not assessed. The median PFS was 48 weeks (95% CI, 43 to 56 weeks). Sixty-nine (83%) out of 83 patients with tumor marker analysis and 63 (75%) out of 84 patients with the same markers had reduced levels of serum calcitonin and carcinoembryonic antigen during treatment compared to baseline, respectively. Diarrhea, fatigue, hypothyroidism, hypertension and anorexia were the most prevalent treatment-related AEs. Except for three *RAS* mutations found in this research study (two in *H-RAS* and one in *K-RAS)*, the majority were found in *RET* (72% of mutation-positive sporadic MTC patients), with the M918T point mutation found in 89% of *RET*-mutation-positive sporadic MTC patients. However, the study only included individuals with advanced illness linked to the M918T *RET* mutation. Motesanib was shown in vitro to suppress wild-type *RET* but not the C634W mutant [129], a prevalent mutation in inherited MTC. However, evidence (including this trial) suggests that motesanib may inhibit the growth of thyroid cancer through pathways other than *RET* activation. Considering only the ORR of this study, the efficacy of motesanib may be questionable. However, the rates of SD and durable SD are quite significant, suggesting its potential for future research.

### 4.2. RET-Specific TKI

Selpercatinib (LOXO-292) is a highly selective RET inhibitor. It was approved by the FDA for NSCLC in adult patients harboring *RET* fusions. Regarding MTC, adults and children ≥ 12 years old with advanced or metastatic *RET*-fusion-positive or *RET* mutant thyroid cancer who require systemic therapy and who are radioactive iodine-refractory have been eligible for treatment with this drug since 2020 [130,131]. Previously, selpercatinib was already tried in a case of advanced metastatic MTC. Subbiah et al. reported one case showing a clinical benefit of selpercatinib vs. MKIs. A patient diagnosed with MTC with lymph node metastasis received a total thyroidectomy, and a genetic analysis of the tumor was performed. A *RET* M918T mutation was detected, and more detailed imaging revealed liver metastases. At this point, TKIs offered a better option for this patient, so he was sequentially treated with six MKIs: sorafenib, vandetanib, cabozantinib, sitravatinib, agerafenib and vandetanib plus everolimus. The patient presented with PD in the liver with large volume ascites and severe fatigue, culminating in these therapeutic choices. LOXO-292 administration was initiated at a dose of 20 mg twice daily, increasing gradually to 160 mg twice daily. This led to significant target inhibition, clinically evaluated as a decrease in serum CEA and calcitonin levels. It induced a radiographic tumor response of 54% (via RECIST) after 6.9 months of treatment. Molecularly, plasma allele fractions encoding *RET* V804M and *RET* M918T in cell-free DNA decreased and remained reduced during the first 8 weeks of treatment. AEs observed during treatment were grade I, and none were linked to drug intake; the patient remained on selpercatinib for more than 7 months [132].

The accumulation of successful cases has led to wider clinical trials exploiting selpercatinib’s potential. Wirth et al. performed a clinical trial with MTC *RET*-mutant and *RET*-fusion-positive thyroid cancer patients, dividing them in two groups: patients who had already received vandetanib or cabozantinib, and patients who were TKI-naïve. Across the three efficacy analysis cohorts, 162 patients were included in the trial: 55 with *RET*-mutant MTC previously treated with vandetanib, cabozantinib or both; 88 with *RET*-mutant MTC not previously treated with vandetanib or cabozantinib; and 19 with previously treated *RET*-fusion-positive thyroid cancers, i.e., 13 patients with the papillary subtype, 3 with poorly differentiated cancer, 2 with anaplastic cancer and 1 with Hürthle cell carcinoma. The primary endpoint was to evaluate the ORR. The percentage of patients previously submitted to vandetanib, cabozantinib or both that were responsive to selpercatinib was 69% (95% CI, 55–81 and the 1-year PFS was 82% (95% CI, 69–90). The ORR was 73% (95% CI, 62–82) in 88 patients with *RET*-mutant MTC who had not been previously treated with vandetanib or cabozantinib, and the 1-year PFS was 92% (95% CI, 82–97). The response rate in 19 patients with previously treated *RET*-fusion-positive thyroid cancers was 79% (95% CI, 54–94), and their 1-year PFS was 64% (95% CI, 37–82). A biochemical response was observed in 91% (95% CI, 80–97) of 54 patients and 66% (95% CI, 52–79) of 53 patients with respect to calcitonin and CEA levels, respectively. Hypertension, increased alanine aminotransferase level, increased aspartate aminotransferase level, hyponatremia and diarrhea were the most prevalent grade 3 or 4 AEs [133].

Baek et al. presented four cases, two of which were *RET*-mutated MTC patients (one germline, D898Y, and another somatic, M918T). Both had metastasis at presentation and received vandetanib as a previous treatment. In one case, PR was detected on a CT scan after six months of selpercatinib therapy, and the patient’s pain from the neck lesion was reduced. An imagological analysis of several other bone metastases (in the sternum, cervical–thoracic spine and bilateral ribs) revealed significantly reduced activity, indicating PR. Serum calcitonin and CEA levels were substantially decreased (from 8541 pg/mL to 52.91 pg/mL and from 886 ng/mL to 247 ng/mL, respectively). At 12 weeks, hypercalcemia and decreased glomerular filtration were detected, leading to the interruption of the treatment. However, once they managed to stabilize the patient, selpercatinib was reintroduced. The treatment led to no setbacks with respect to hypercalcemia and the glomerular filtration rate, so, the connection between selpercatinib and these AEs was possibly not a direct link. The other case, harboring an M918T somatic mutation, revealed PD with liver metastasis and blurred vision between the discontinuation of vandetanib and the initiation of selpercatinib treatment. After a detailed examination, choroidal metastasis was detected. Selpercatinib was initially administered at 120 mg twice a day, and in one month, PR was achieved on the choroidal tissue. Regarding the liver metastasis, there was an observable decrease in size. As in the first case, the calcitonin and CEA levels improved (from 944.6 ng/mL to 12.19 ng/mL and from 13.7 ng/mL to 9.32 ng/mL, respectively) [134].

Zeteletinib (BOS172738) is a RET-specific inhibitor that recently arose as an option. Its difference from other selective RET inhibitors is that it also inhibits VEGFR2 (with >300-fold sensitivity), maximizing the treatment’s effect on tumor growth. Schoffski et al. performed a clinical trial (phase I) with 67 patients with *RET*-altered advanced tumors (16 MTC and 51 NSCLC cases). The ORR was 44%, and one patient achieved CR. The AEs were mostly grade 2 or even lower and were not entirely connected to the treatment. Interestingly, hypertension, which is the most common AE associated with TKIs, had a low rate of incidence [135].

Pralsetinib (BLU-667) is a potent selective RET inhibitor that has received significant attention in recent years. Subbiah et al. biochemically described this drug and compared it to MKIs with respect to its half-maximal inhibitory concentration IC50 (Table 2) [136]. Pralsetinib inhibited wild-type RET 27.5 times more than cabozantinib and 10 times more than vandetanib. However, regarding VEGFR2, cabozantinib is 17.5 times more potent than pralsetinib, and vandetanib is 8.75 times more potent than pralsetinib.

To test pralsetinib’s biological behavior, cells genetically engineered to express *KIF5B–RET* fusion were treated with pralsetinib and another MKI for comparison [136]. Pralsetinib inhibited the RET pathway 10 times more efficiently than vandetanib, cabozantinib and agerafenib (RXDX-105). The degree of inhibition was tested for each signaling pathway. Pralsetinib significantly decreased the levels of transcripts deriving from all pathways except GSK3B, a transcript from the PI3K/AKT/mTOR pathway. In vivo xenografted MTC models equally demonstrated the potent activity of pralsetinib toward *RET* alterations. Considering these results and acknowledging BLU-667’s potential, clinical trials were performed to obtain real-time data proving the efficiency and efficacy of this drug. In this same study, a sporadic MTC case was presented: a female, TKI-naïve patient with multiple *RET* mutations and multiple metastases (lung, liver, esophagus and trachea). Calcitonin was reduced by more than 90% following 28 days of BLU-667 treatment. The patient experienced PR with a tumor decrease of >30% after repeated increases in the dosage of BLU-667 to 200 mg once day, according to RECIST version 1.1. At 10 months, this patient’s dose was increased to 300 mg BLU-667 once daily, and the patient obtained PR (47% maximum decrease). CEA levels declined by 57% overall throughout this time period. The patient remained progression-free and on therapy for more than 11 months. Other novel results were presented with in this study, including in one patient who harbored an M918T mutation that presented circulating tumor DNA, which decreased by around 47% after 28 days and was not detectable after 56 days. The tumorigenic RET pathway was inhibited as well, and it achieved a maximum reduction PR of 47% [136]. In a different trial, 79 patients with different *RET* mutations were treated with pralsetinib. The overall ORR was 65% (95% CI, 53–75; n = 51/79.5% CR, 59% PR; 1 pending confirmation). For patients who were previously treated with cabozantinib and/or vandetanib, the ORR was 60% (95% CI, 46–74; n = 32/53; 2% CR; 58% PR; 1 pending), while it was 74% (95% CI, 49–91; n = 14/19; 5% CR; 68% PR; all confirmed) for patients who had never received therapy. The DCR was 97% (95% CI, 91–100), and 99% (78/79) of patients had a reduction in tumor size. The medians for both the duration of response and PFS were not disclosed [137]. Table 3 includes the main outputs and the most common adverse events in TKIs.

### 4.3. Immunotherapy

At present, immunotherapy stands as an emerging therapeutical option. Immunotherapy, particularly the use of checkpoint inhibitors targeting programmed death-ligand 1 (PD-L1), has shown potential as a therapeutic tool for MTC [138]. PD-L1 expression is being considered a potential biomarker of response to anti-PD-1 or anti-PD-L1 agents in PD-L1 positive tumors [138,139]. Clinical trials demonstrated the efficacy of PD-1/PD-L1 immunotherapies in improving therapeutic response [138]. There are recent studies focusing on blocking the PD-1/PD-L1 interaction in MTC patients [140,141,142,143]. Yamamoto et al. were the first to perform a clinical trial using nivolumab in one MTC patient. This patient reached PR for >12 months [142]. Lorch et al. explored how nivolumab and ipilumab can be used in MTC since the expression of CTLA-4 is also detected in MTC patients [144]. However, none of the MTC patients developed PR throughout the trial [145].

Another therapeutical strategy was reported by Schott et al. using dendritic cell vaccination [143]. They have performed a trial in seven MTC patients, injecting them with dendritic cells loaded with CEA and calcitonin peptides. Throughout 13.1 months, all patients developed a hypersensitive skin reaction caused by perivascular and epidermal infiltration of CD4^+^ memory T cells and CD8^+^ cytotoxic T cells. One patient showed a 36% decrease in calcitonin levels in the first 2 months, but due to a progression in liver metastases after 10 months, immunotherapy was discontinued, and chemotherapy was initiated. Another patient developed partial tumor remission and after 12 vaccinations, immunoreactivity toward CEA peptide and calcitonin peptide was observed, confirming a T cell activation response against both antigens. The authors consider that this treatment may be important not only to MTC but other endocrine malignancies [143].

Chatal et al. suggested a new option for advanced-stage MTC patients: radioimmunotherapy [146]. The authors studied a series of 29 patients with metastatic MTC and low calcitonin doubling times (<5 years, associated with a high degree of aggressiveness of the tumor). These patients were submitted to radioimmunotherapy using an anti-CEA monoclonal antibody and ^131^I-labeled hapten carrying the therapeutic payload. The treated group did not show statistically different OS compared to the control group, which comprised MTC patients without treatment. However, the subset of patients with a calcitonin doubling time of less than 2 years revealed an increase in OS compared to the control group. This treatment led to notable AEs: more than 20% of patients had grade 4 neutropenia or thrombocytopenia, and one patient developed myelodysplastic syndrome [146].

Table 4 summarizes these trials according to the authors’ observations and the AEs reported. Despite the recent appearance of trials testing new immune checkpoints in MTC, the majority are still not approved by FDA for MTC treatment. Table 4 summarizes immune checkpoints and the inhibitors studied in two clinical trials for MTC treatment.

### 4.4. Somatostatin Receptor (SSTR) Inhibitors

One of the emerging treatment approaches for MTC is the use of somatostatin receptor (SSTR) inhibitors. Somatostatin is a naturally occurring hormone that regulates various physiological processes in the body. It acts through specific receptors called SSTRs, which are found on the surfaces of various cells, including neuroendocrine cells, and therefore MTC cells. These receptors play a role in modulating cell growth, hormone secretion and angiogenesis [148]. Within SSTR family, Herac et al. studied possible correlations between SSTR2A and SSTR5 expression and MTC aggressiveness [149]. A positive correlation was found between SSTR2A expression and the appearance of lymph node metastasis, but the same was not significant for SSTR5. Both SSTRs were correlated with advanced stages of disease and desmoplasia, revealing that high levels of SSTR2A and SSTR5 expression can be poor prognostic features [149]. This association is the origin of new drugs aiming to inhibit SSTRs. SSTR inhibitors are synthetic analogs of somatostatin that were developed to target and bind to SSTRs on MTC cells. By binding to these receptors, SSTR inhibitors exert several effects that can be beneficial in the treatment of MTC, including the inhibition of calcitonin secretion and the downregulation of some pathways, e.g., MAPK and PI3K/AKT [150].

Giardino et al. studied the effects of two SSTR inhibitors, octreotide and pasireotide, on a TT cell line. Both drugs reduced tumor cell proliferation, MAPK activation and cell migration [151]. Lanreotide (LAN) is another SSTR inhibitor that was tested both in vitro and in vivo. Vitale et al. demonstrated in a TT cell line that cytotoxicity was increased more in cells exposed to both LAN and IL-2 than in cells without treatment and in a series of six patients, after 6 months of therapy, two achieved partial response and three reached stable disease, with significant decreases in calcitonin levels [152]. Table 5 summarizes two clinical trials using octreotide and lanreotide for MTC treatment.

## 5. Discussion

Personalized cancer medicine falls under the umbrella of a tailored approach that uses an individual’s genotype to identify the most effective approach for disease prevention, screening and treatment. The main driver in the last decade has been the capacity and availability of high-throughput technologies, mainly next-generation sequencing, and the generation of a vast amount of genetic cancer information. In this scope, efforts have been made to establish associations between patients’ molecular characteristics and their responses to drug treatments or survival, and ideally to minimize the side effects caused by conventional options. Personalized cancer medicine involves predicting the likelihood of developing cancer and selecting screening techniques to minimize the risk and testing burden, matching patients with more effective treatments that have fewer side effects and predicting the chances of cure and recurrence. While the development and improvement of chemotherapy transformed cancer treatment, many patients still do not respond to treatment or eventually become resistant to therapies. MTC treatment, mainly in advanced disease, represents a fine example of how targetable genetics events, namely, *RET* alterations, can guide personalized treatment decisions. As presented, patients with *RET* mutations may be eligible for targeted therapies like vandetanib or cabozantinib, MKIs that inhibit the abnormal RET signaling pathway, and other upstream receptors commonly involved in MTC. Selpercatinib and pralsetinib, which are RET-specific TKIs, are eligible for use in patients with advanced-stage MTC and harbor *RET* mutations or *RET* fusions, despite the latter being rare. These examples highlight how genotyping in MTC enables personalized medicine by guiding treatment decisions, identifying potential therapeutic targets and predicting treatment response. Still, there is a need for improvement as the initial TKIs only demonstrated moderate success but exposed toxicity as a non-neglectable concern [154]. To improve the clinical success of treating these patients and to reduce the robust adverse events, there are novel TKIs already available. The new generation, instead of targeting many growth factors and receptors, which were termed “dirty” in the past, now present high levels of specificity. *RET*-specific inhibitors are demonstrating promising results, improving valuable parameters such as efficacy, efficiency and decreased toxicity compared to MKIs. These are not exclusive to MTC, as in other *RET*-altered neoplasia there is already greater experience with promising outcomes. The ongoing clinical debate, will now be to understand whether MTC patients should be treated with MKI or *RET*-specific inhibitors, to which genotypes the response will be more efficient and what the timing of therapy administration should be, i.e., if we should anticipate them and consider them as first-line options. The connection between a patient’s genotype and the best therapeutical option and reducing resistance acquisition and AEs is still limited; however, this data, in particular the better responses of vandetanib and cabozantinib to the presence of a M918T mutation, already reinforce the need to establish this correlation in the future, particularly for *RET* and *RAS* mutations.

One major concern nowadays remains the strong possibility of resistance acquisition. Rosen et al. described how the long-term use of selpercatinib could transform cells biologically in order to acquire resistance [155]. MAPK reactivation, PI3K alterations, additional *RET* mutations, *K-RAS* mutations, *BRAF* mutations and *MET* or *FGFR1* amplifications are among the resistance mechanisms observed in tumor cells during this study, and several of these mechanisms are often observed in the same patient, suggesting that polyclonal resistance may occur regularly [155]. Taking into consideration that MAPK and other pathways are not direct targets of *RET*-specific TKIs, it may be applicable in these cases, restoring MKIs to counteract treatment resistance and importantly, to evaluate if this combination is not antagonizing and causing unmanageable adverse events [155].

## 6. Conclusions

The use of TKIs is a valuable option for the treatment of MTC patients with advanced stages of disease. Based on previous experience, we have learned that we may encounter resistance mechanisms and significant adverse effects that cannot be ignored. We report a wide number of clinical trials investigating different therapeutical options and how they can complement each other. A new era is dawning with the introduction of *RET*-specific TKIs that offer greater pharmacological efficacy and selectivity. We must now understand what the perfect combination of these drugs is that will maximize the outcomes for the patients’ benefit.

In the context of personalized medicine, the minimal genetic complexity of MTC and its enrichment in the prevalence of *RET* mutations positions this disease as an ideal candidate for a success story. The rapid advancement of targeted therapies has promise to reduce the impact of this disease, ultimately aiming to bring it under control or ideally, achieve a cure state.

## Figures and Tables

**Table 1:** Clinical trials using MKIs in MTC. The table includes the form of action, the main outputs, most common adverse events and the study reference.

Tyrosine Kinase Inhibitor	Target of Inhibition	PFS	ORR	Observations	Most Common Adverse Events	Ref.
Vandetanib *	VEGFR2-3 EGFR EGFR2 RET MMPs	30.5 mo.	46.4% (hereditary) 51.8% (sporadic)	37% PD and 15% had died. OS was immature at data cutoff.	Diarrhea, rash, nausea, hypertension and headache	[90]
Cabozantinib *^&^	MET VEGFR2 RET KIT FLT-3	11.2 mo.	28%	100% PR in cabozantinib group	Diarrhea, palmar–plantar erythrodysesthesia (hand–foot syndrome), fatigue and weight loss	[104]
**Sorafenib ^&^**	VEGFR2 PDGFR RAF1 KIT FLT-3 RET BRAF	17.9 mo.	NR	6.3% PR; 87.5% SD regarding cabozantinib group	Diarrhea, hand–foot–skin reaction, rash and hypertension	[105]
**Sunitinib**	VEGFR1-3 PDGFR KIT FLT-3 CSF-1 RET	10.6 mo.	50%	25% SD; 25% PD. Chemotherapy patients only presented with 3.5 mo. PFS.	Fatigue, hypertension, dysgeusia and cutaneous papules	[106]
**Lenvatinib ^&^**	VEGFR1-3 PDGFRα FGFR1-4 KIT RET	18.3 mo.	64.8%	4 patients CR; 165 patients PR	Hypertension, diarrhea, fatigue or asthenia, decreased appetite, weight loss and nausea	[107]
**Anlotinib**	VEGFR1-3 EGFR FGFR1-4 PDGFR KIT	17.5 mo. & 20.7 mo.	48.4%	88,7% DCR; 17.5 mo. PFS for patients older than 50 y/o; 20.7 mo. PFS for bone metastases	Hypertension, palmar- plantar erythrodysesthesia syndrome, proteinuria, hypertriglyceridemia, QTc prolongation, diarrhea and fatigue	[108]
**Axitinib**	VEGFR PDGFR KIT	9.4 mo.	23.1%	0% CR; 23.07% PR; 38.46% SD; 30.77% PD; 1 patient NR. Best results achieved as first-line treatment.	Diarrhea, hypertension, mucositis and weight loss	[109]
**Pazopanib**	VEGFR PDGFR KIT FGFR	9.4 mo.	15%; 20%; and 10%	19.9 mo. OS; ORR: 15% without prior treatment; 20% in TKI naïve and 10% in prior TKI treatment patients; 1 death after withdrawal, potentially treatment related	Hypertension, fatigue, diarrhea and abnormal liver tests	[110]
**Motesanib**	VEGFR PDGFR KIT RET WT	48 weeks	2%	81% SD	Diarrhea, fatigue, hypothyroidism, hypertension and anorexia	[111]

* FDA- and EMA-approved for MTC. ^&^ FDA- and EMA-approved for radioactive iodine-refractory differentiated thyroid cancer. PR: partial response; CR: complete response; SD: stable disease; DCR: disease control rate; PD: progressive disease; PFS: progression-free survival; OS: overall survival; ORR: objective response rate; NR: not reached; QTc: measurement of the time between the start of the Q wave and the end of the T wave on an electrocardiogram waveform.

**Table 2:** Comparison between *RET*-specific tyrosine kinase inhibitors (TKIs) and MKIs with respect to half-maximal inhibitory concentration (IC50); lower IC50 values represent higher levels of toxicity to that target.

Tyrosine Kinase Inhibitor	Biochemical Target	IC50 (nmol/L)
**Pralsetinib**	WT RET	0.4
	RET M918T	0.4
	VEGFR2	35
**Cabozantinib**	WT RET	11
	RET M918T	8
	VEGFR2	2
**Vandetanib**	WT RET	4
	RET M918T	7
	VEGFR2	4

**Table 3:** Clinical trials using RET-specific tyrosine kinase inhibitors in MTC.

Tyrosine Kinase Inhibitor	PFS	ORR	Observations	Most Common Adverse Events	Ref.
**Selpercatinib**	92%	73%	PFS was attained in percentage; 91% had calcitonin decrease and 66% had CEA reduction; at 1 year, 64% remained progression-free.	Hypertension, increased aspartate and alanine aminotransferase, hyponatremia and diarrhea	[133]
**Zeteletinib**	NR	44%	ORR was higher in MTC vs. NSCLC patients (44% vs. 30%).	Creatinine phosphokinase increase, dyspnea, aspartate aminotransferase increase, diarrhea, anemia and fatigue	[135]
**Pralsetinib**	NR	65%	97% DCR; 99% had tumor size reduction; 75% remained on treatment.	Aspartate and alanine aminotransferase increased, anemia, hypertension and constipation	[137]

DCR: disease control rate; PFS: progression-free survival; ORR: objective response rate; NR: not reached; MTC: medullary thyroid carcinoma; NSCLC: non-small cell lung carcinoma; CEA: carcinoembryonic antigen.

**Table 4:** Two clinical trials using PD-1/PD-L1 and CTLA-4 immune checkpoints as targets of inhibition in MTC.

Immune Checkpoints	Immunotherapy Drugs	Observations	Most Common Adverse Events	Ref.
**PD-1/PD-L1**	Nivolumab	PR for >12 months	Ventricular extrasystoles, constipation, diarrhea and fatigue.	[142]
**CTLA-4**	Nivolumab + Ipilumab	Considerable activity in ATC, but no response was observable in MTC.	NR	[147]

PR: partial response; MTC: medullary thyroid carcinoma; ATC: anaplastic thyroid carcinoma; NR: not reached.

**Table 5:** Clinical trials using SSTR inhibitors.

SSTR Inhibitors	Observations	Most Common Adverse Events	Ref.
**Octreotide**	Octreotide does not improve the natural course of advanced stages of medullary thyroid carcinoma.	Diarrhea and weight loss.	[153]
**Lanreotide**	After 6 months of therapy, partial response and stable disease have been recorded in 2 and 3 patients, respectively, with significant decreases in calcitonin levels in 3 patients.	Diarrhea, weight loss and fatigue.	[152]

## Data Availability

Not applicable.
